# Effects and feasibility of a standardised orientation and mobility training in using an identification cane for older adults with low vision: design of a randomised controlled trial

**DOI:** 10.1186/1472-6963-9-153

**Published:** 2009-08-27

**Authors:** GAR Zijlstra, GHMB van Rens, EJA Scherder, DM Brouwer, J van der Velde, PFJ Verstraten, GIJM Kempen

**Affiliations:** 1 Maastricht University, Faculty of Health, Medicine and Life Sciences, Department of Health Care and Nursing Science, Maastricht, the Netherlands; 2 CAPHRI School for Public Health and Primary Care, P.O. Box 616, 6200 MD Maastricht, the Netherlands; 3 VU University Medical Center, Department of Ophthalmology, and the Institute for Research in Extramural Medicine (EMGO), P.O. Box 7057, 1007 MB Amsterdam, the Netherlands; 4 University of Groningen, Institute of Human Movement Sciences, P.O. Box 72, 9700 AB Groningen, and VU University Amsterdam, Van der Boechorststraat 1, 1081 BT Amsterdam, the Netherlands; 5 Bartiméus, Institute for the Blind and Visually Impaired, Oudenoord 325, 3513 EP Utrecht, the Netherlands; 6 Rotterdam University of Applied Sciences, School of Healthcare Studies, Museumpark 40, 3015 CX Rotterdam, the Netherlands; 7 Royal Visio, National Foundation for the Visually Impaired and Blind, P.O. Box 1180, 1270 BD Huizen, the Netherlands; 8 Sensis – Organisation for Blind and Partially Sighted People, Department of Innovation and Expertise, P.O. Box 54, 5360 AB Grave, the Netherlands

## Abstract

**Background:**

Orientation and mobility training (O&M-training) in using an identification cane, also called symbol cane, is provided to people with low vision to facilitate independent participation in the community. In The Netherlands this training is mainly practice-based because a standardised and validly evaluated O&M-training in using the identification cane is lacking. Recently a standardised O&M-training in using the identification cane was developed. This training consists of two face-to-face sessions and one telephone session during which, in addition to usual care, the client's needs regarding mobility are prioritised, and cognitive restructuring techniques, action planning and contracting are applied to facilitate the use of the cane. This paper presents the design of a randomised controlled trial aimed to evaluate this standardised O&M-training in using the identification cane in older adults with low vision.

**Methods/design:**

A parallel group randomised controlled trial was designed to compare the standardised O&M-training with usual care, i.e. the O&M-training commonly provided by the mobility trainer. Community-dwelling older people who ask for support at a rehabilitation centre for people with visual impairment and who are likely to receive an O&M-training in using the identification cane are included in the trial (N = 190). The primary outcomes of the effect evaluation are ADL self care and visual functioning with respect to distance activities and mobility. Secondary outcomes include quality of life, feelings of anxiety, symptoms of depression, fear of falling, and falls history. Data for the effect evaluation are collected by means of telephone interviews at baseline, and at 5 and 17 weeks after the start of the O&M-training. In addition to an effect evaluation, a process evaluation to study the feasibility of the O&M-training is carried out.

**Discussion:**

The screening procedure for eligible participants started in November 2007 and will continue until October 2009. Preliminary findings regarding the evaluation are expected in the course of 2010. If the standardised O&M-training is more effective than the current O&M-training or, in case of equal effectiveness, is considered more feasible, the training will be embedded in the Dutch national instruction for mobility trainers.

**Trial registration:**

ClinicalTrials.gov NCT00946062

## Background

Low vision is common among older people and is associated with mobility problems, limitations in physical and social functioning, ADL and IADL disability, reduced well-being, falls, depression, loneliness and mortality e.g. [1-15]. Particularly the level of mobility is essential for conducting activities in daily life, but this domain of functioning is directly affected by visual impairments [11,12,15,16]. Mobility problems – as a result of visual impairments – may therefore threaten independent functioning of older persons.

Mobility problems in older persons are related to a series of negative outcomes. Limitations in mobility are found to be associated with lower levels of quality of life, depression, cognitive dysfunctioning, fair or poor health, hip fracture, disability, institutionalisation and mortality [7,17-22]. Mobility limitations as a result of visual impairments may particularly limit a person's ability to travel independently and, consequently, many aspects of daily routine life as well as life satisfaction are affected substantially [11,23-27]. For participating in social and physical activities, abilities for travelling are crucial. Travel in the environment involves skills of orientation and mobility. Orientation is the ability to recognize the environment and establish position in relation to the environment, whereas mobility is the physical ability to move in an orderly, efficient, and safe manner through the environment [28]. To maintain travel independence, it is essential for a visually impaired person to learn new orientation and mobility skills to compensate for reduced visual information.

Orientation and mobility training (O&M-training), which is a component of the rehabilitation facilities for visually impaired people, aims to maintain independence of travel by teaching visually impaired persons to ambulate and negotiate the environment safely and independently. It may decrease mobility limitations and contribute positively to societal participation and quality of life. O&M-training is often supplemented by the use of assistive devices [29]. Common mobility devices that have been used in O&M-training among persons with visual impairments are canes, such as the identification cane and the long cane [28]. With training in the use of visual and non-visual information including assistive devices, visually impaired persons gain a better understanding of their environment, which enables them to travel more comfortable, efficiently, and safely. Early intervention might be essential to slow down the progression of disablement [30], as persons with visual impairment are particularly at risk of developing disability [29,31,32]. Although the benefits of O&M-training have been object of study, the conducted evaluation studies have substantial limitations such as the lack of a control group [33,34] or randomisation [28], or the inclusion of rather small study populations [28,35]. Furthermore, none of these studies focussed on other societal outcomes next to mobility such as (social) participation and quality of life. Assessing multiple outcomes in effect studies are, however, recommended since Crews and colleagues indicated that interventions in the area of visual impairments may have effects on one kind of outcomes, such as mobility, but not on others, such as quality of life [36].

In The Netherlands, no previous research on the impact of O&M-training on daily functioning in older people with visual impairments has been conducted. In addition, evaluation studies of O&M-training in the use of an identification cane, also called symbol cane, which is frequently part of rehabilitation care for adults with low vision in The Netherlands, were hardly identified in the international literature. And finally, although there is a Dutch national instruction on orientation and mobility for mobility trainers employed in low vision rehabilitation care, there is no standardised protocol in delivering such O&M-training to older adults with low vision.

### Objectives of the study and the current paper

The main objectives of the study are:

1) to develop a standardised O&M-training in the use of an identification cane by older adults with low vision;

2) to evaluate this newly developed standardised training with respect to effectiveness and feasibility in a randomised controlled trial.

The current paper presents the design of the randomised controlled trial that will evaluate the standardised O&M-training in the use of the identification cane by older adults with low vision in The Netherlands.

## Methods/Design

### Study design and setting

A parallel group randomised controlled trial was developed to evaluate a standardised O&M-training in the use of an identification cane (NCT00946062). The trial is conducted in collaboration with the two main organisations for low vision rehabilitation care in The Netherlands: Bartiméus and the VisioSensisDeBrink Group (as of January 2010: Royal Visio). In 18 local centres, which are scattered over The Netherlands, O&M-training is provided to older persons with visual impairment. The centres were randomly allocated to the control group or intervention group stratified by organisation (at that time: Bartiméus – four local centres, Sensis – four local centres, and Visio – ten local centres).

**Figure 1:**
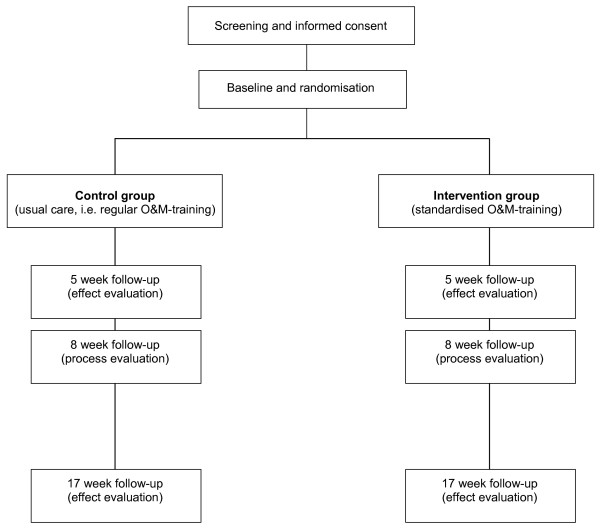
**Design of the trial**.

To screen eligible people for the trial, the local centres provide the research team with information on older clients with low vision who may receive mobility support. After the screening procedure and baseline measurement clients in the control group receive usual care, i.e. the regular O&M-training in using the identification cane, and clients in the intervention group receive the standardised O&M-training in using the identification cane. Follow-up data for the effect evaluation are collected at 5 and 17 weeks after the start of the training by means of telephone interviews. To obtain data for the process evaluation trainers complete registration forms after each training session and clients are interviewed by telephone at 8 weeks after the start of the training. Figure 1 shows the design of the trial. The Medical Ethical Committee of the Maastricht University/Academic Hospital Maastricht granted approval for conducting this trial.

### Study population

The screening of potential participants started in November 2007 and occurs in a stepped procedure. *Firstly*, during the local centre's standard exploratory interview a staff member of a local centre roughly screens a potential participant using a six-item pre-structured registration form. The items include whether the client: (1) suffers from low vision, (2) is 55 years of age or older, (3) lives independently or in a home for older people, (4) experiences difficulty to avoid large obstacles due to the vision loss, (5) suffers from additional impairments that cause full inability to leave the home, and (6) consents to an additional screening interview by telephone for potential participation in the trial. If items 1, 2, 3 and 6 are scored 'yes' and items 4 and 5 are scored 'no', contact information is recorded and the registration form is sent to the research team. *Secondly*, a pre-structured 10-minute interview by telephone is conducted by the research team to determine the client's eligibility for participation in the trial. The additional inclusion criteria are: 1) able to go outside for a short walk or doing groceries, and 2) experiencing difficulties with safely crossing a street, or experiencing difficulties with recognising acquaintances outdoors, or willing to become recognisable as partially sighted by means of the identification cane. *Lastly*, mobility trainers decide during an individual contact with the client whether O&M-training in the use of an identification cane is the eligible care for the client.

Clients are excluded if one of the following criteria is met: a) cognitive impairment (a score of less than 4 on the Abbreviated Mental Test 4 (AMT4) during an interview by telephone) [37,38], b) unable to complete an interview by telephone due to language or hearing problems, c) unable to participate in or finish the O&M-training due to confinement to bed or possible nursing home admission, d) permanent use of a walking aid that is incompatible with the use of an identification cane, e) having recently received an O&M-training in the use of an identification cane and permanent use of this cane outdoors, and f) not receiving an O&M-training in the use of an identification cane as treatment for their mobility problem.

Eligible clients receive oral and written information about the trial during the first and the second step of the screening procedure. Those clients who signed an informed consent form were included in the trial. An overview of all inclusion and exclusion criteria is shown in Table 1. To maintain active participation of the local centres, the staff receives regular updates with respect to the number of clients and their progress in the trial by means of newsletters, monitoring telephone contacts and weekly reviews by email.

**Table 1:** Screening for participants: inclusion and exclusion criteria

**Inclusion criteria:**	**Exclusion criteria:**
- Aged 55 years or over	- Cognitive impairment (a score of less than 4 on the Abbreviated Mental Test 4) [37,38]
- Low vision	- Language or hearing problems that impede completing an interview by telephone
- Living independently in the community or in a home for older people	- Confinement to bed or possible nursing home admission that impede completion of the O&M-training
- Able to see large obstacles and to go outside for a short walk or doing groceries	
- One of the following:	
▪ experiencing difficulties with safely crossing a street	- Permanent use of a walking aid incompatible with the use of an identification cane
▪ experiencing difficulties with recognising acquaintances outdoors	- Having recently received an O&M-training in the use of an identification cane and permanent use of this cane
▪ willing to become recognisable as being partially sighted by using the identification cane	
- Written informed consent	
- Orientation and Mobility training (O&M-training) in the use of an identification cane	

### Randomisation and masking

After completing a baseline interview by telephone and confirmation on the eligibility of the O&M-training in the use of an identification cane by the mobility trainer, clients are officially assigned to the regular or the standardised O&M-training. To prevent contamination bias randomisation stratified by organisation was performed at the level of the local centres. Randomisation at the client or mobility trainer level may lead to contamination because (1) a mobility trainer may then train clients in the control group as well as clients in the intervention group or (2) a mobility trainer may unintentionally inform other mobility trainers about the standardised O&M-training due to discussing client and training progress at shared workspaces and during meetings. In addition, randomisation at the level of the mobility trainers was considered impractical by the organisations, because most mobility trainers are allocated to a defined district to limit travelling time within the region of their local centre.

The collaborating organisations for low vision rehabilitation care in The Netherlands, i.e. Bartiméus and VisioSensisDeBrink Group, consented with participation in the trial before randomisation of the local centres. To control for differences in organisation characteristics, randomisation of local centres was performed per organisation (at that time: Bartiméus, Sensis and Visio). To assure even distribution of local centres with respect to expected number of clients per month and to avoid contamination due to close collaboration of several local centres, all local centres per organisation were matched in pairs: Bartiméus – four local centres, one pair; Sensis – four local centres, two pairs, and; Visio – ten local centres, three pairs. According to a predefined format one of each pair was randomly allocated to the intervention group or control group. This was carried out by an external party who threw a dice; even numbers corresponded to allocation to the intervention group and odd numbers to allocation to the control group.

Due to the nature of the trial and the information provided to clients before the start of the trial (required by the Medical Ethical Committee), mobility trainers, researchers and participants are not masked to intervention status. Participants are, however, not informed about their intervention status until the start of the O&M-training. Trained outcome assessors who perform the interviews by telephone are masked for intervention status.

### Orientation and mobility training

Despite the presence of a Dutch national instruction on orientation and mobility for mobility trainers employed in low vision rehabilitation care, there is no fixed protocol for O&M-training in the use of an identification cane. To obtain insight into this O&M-training as provided by the mobility trainers, information was collected between March and June 2007 by means of: 1) observing O&M-training sessions (n = 5), 2) studying the literature of the Dutch national instruction on orientation and mobility and attending its session on the identification cane, and 3) performing individual face-to-face interviews with mobility trainers (n = 18) of the organisations for rehabilitation care for people with visual impairment in The Netherlands. The interviews were guided by a questionnaire. In addition to questions related to the characteristics of the current O&M-training in the use of an identification cane (see Table 2), mobility trainers were asked to reflect on elements missing in the current O&M-training and potential opportunities to improve this training. Based on information obtained from the mobility trainers and the national instruction on orientation and mobility, a new standardised O&M-training in using the identification cane was developed.

**Table 2:** Characteristics of the regular and standardised O&M-training

**Characteristics**	Regular O&M-training*	**Standardised O&M-training**
Elements:	1. Exploration of client's needs^t^	1. Exploration of client's needs^t^
	2. Providing information, e.g. on walking aids, canes and techniques related to orientation and safe behaviour^t^	2. Prioritising the client's needs^c^
	3. Training techniques - related to orientation and safe behaviour -outdoors while using the identification cane^t, c^	3. Providing information, e.g. on walking aids, canes and techniques related to orientation and safe behaviour^t^
		4. Formulating, performing and evaluating action plans^c^
		5. Training techniques - related to orientation and safe behaviour -outdoors while using the identification cane^t, c^
		6. formulating action plans/contracting^c^

Applied techniques:	- (Unknown)	- Prioritising client's needs
		- Cognitive restructuring
		- Action planning
		- Contracting
		- Providing direct feedback
		- Stimulating individual problem solving/finding realistic solutions

Number of sessions:	Variable, mostly 1-2 sessions (range 1-5)	3 sessions

Frequency:	Variable, mostly weekly (if multiple sessions were conducted)	Every other week

Duration:	- Complete training time:	- Complete training time:
	▪ variable (range 60-120 min)	▪ session 1: 90 min
		▪ session 2: 80 min
		▪ session 3: 25 min
	- Training time indoors:	- Training time indoors:
	▪ variable (range 15-60 min)	▪ session 1: 60 min
		▪ session 2: 40 min
		▪ session 3: 25 min

Format of sessions:	Face-to-face	Session 1 and 2: face-to-face
		Session 3: telephone contact

Location of session:	Variable, mostly client's home environment	Client's home environment

* Based on information obtained during face-to-face interviews with mobility trainers (n = 18).

^t ^Active role for trainer; ^c ^active role for client.

The standardised O&M-training aims to facilitate safe and independent participation in the community by optimal use of one's abilities, and to facilitate uptake of old or new activities. To achieve these aims several strategies were added to the regular O&M-training, that is: prioritising the client's needs, cognitive restructuring, action planning, contracting, providing direct feedback and stimulating individual problem solving, and finding personal, realistic solutions. The standardised O&M-training consists of two face-to-face sessions and one telephone session. Compared to the regular O&M-training, the standardised O&M-training is well structured, yet still tailor-made as clients are actively involved in their rehabilitation with respect to mobility. For example, they are encouraged to individualise their training by means of formulating personal goals regarding activities in the community. Table 2 presents the main characteristics of the regular and standardised O&M-training. More information with respect to the development of the standardised O&M-training will be presented elsewhere.

Mobility trainers were eligible if they provided O&M-training in the use of an identification cane to partially sighted clients of one of the organisations. Trainers who would provide the standardised O&M-training to the intervention group received a two-hour instruction by the researcher (GZ). To prepare for the instruction, trainers read the manual of the standardised O&M-training. After the instruction, mobility trainers informed the researcher if difficulties in applying the standardised O&M-training were observed or questions arose. To monitor whether participants received sufficient care, trainers answered two questions as part of the process evaluation. These questions included to what extent the O&M-training met the participant's need for mobility support according to the perception of the trainer and whether additional training sessions in the use of the identification cane were needed. Trainers were instructed to provide additional O&M-training sessions to the clients if needed. Trainers providing care according to the standardised O&M-training needed to complete both the 2 face-to-face sessions and the one telephone session before conducting extra training sessions on O&M.

No restrictions were held with respect to receiving care other than O&M-training in the use of the identification cane. In The Netherlands the costs of the O&M-training are reimbursed; the costs associated with the purchase of the cane are paid by the consumers.

### Measures

#### Effect evaluation

The concepts measured to determine the effectiveness of the standardised O&M-training comprise to a great extent the 'Activity and Participation' domains of the International Classification of Functioning, Disability and Health [ICF; [39]]. As ICF domains we include 'self care', 'mobility', 'domestic life', 'interpersonal interactions and relationships', and 'community, social and civic life', respectively. An overview of the main outcomes, the number of items, the interpretation of the scoring and the measurements during the course of the study is presented in Table 3.

**Table 3:** Outcome measures of the effect evaluation

**Variable**	**No. of items**	Range*
**Primary outcome measures**		
ADL self care (ADL subscale of the GARS) [40]	11	**11 **to 44
Distance activities and mobility (subscale of the VFQ) [41,42]	8	**8 **to 40

**Secondary outcome measures**		
Activities index (FAI) [43,44]	15	15 to **60**
Social support interactions SSL12-I [45]	12	12 to **48**
Feelings of loneliness [46]	1	1 to **6**
Health-related quality of life (EQ5D) [47]	5	**5 **to 15
Mental health and dependency (subscale of the VFQ) [41,42]	6	**6 **to 30
Feelings of anxiety (HADS-A) [44,48]	7	**0 **to 21
Symptoms of depression (HADS-D) [44,48]	7	**0 **to 21
Concerns about falling (FES-I) [49,50]	16	**16 **to 64
Fear of falling [46]	1	**1 **to 5
Activity avoidance due to fear of falling [46]	1	**1 **to 5
Number of indoor falls in the previous 6 months^† ^[46]	1	**1 **to 6
Number of outdoor falls in the previous 6 months^† ^[46]	1	**1 **to 6

* The bold scores indicate the most favourable scores.

^† ^At FU1 and FU2 the number of falls between the previous and the current assessment is measured.

ADL = activities of daily life; GARS = Groningen Activity Restriction Scale; VFQ = Visual Functioning Questionnaire; EQ5D = EuroQol 5 Dimensions; HADS-A = anxiety subscale of the Hospital Anxiety and Depression Scale; HADS-D = depression subscale of the Hospital Anxiety and Depression Scale; FAI = Frenchay Activities Index; FES-I = falls efficacy scale international.

The primary outcomes of the effect evaluation are *ADL self care*, as assessed by the 11-item ADL subscale of the Groningen Activity Restriction Scale (GARS) [40], and the subscale *distance activities and mobility *of the Visual Functioning Questionnaire (VFQ) [41,42]. The secondary outcomes comprise *domestic, community, and social and civic life *assessed by the subscales domestic, outdoor activities and leisure/work, respectively, of the Frenchay Activities Index (FAI) [43,44], and *interpersonal interactions and relationships*, assessed as social support interactions by the Social Support List (SSL12-I) [45] and as feelings of loneliness by a one-item question (according to Zijlstra and colleagues [46]). Health-related *quality of life *is assessed by the EQ-5D that comprises 5 items on mobility, self-care, usual activities, pain/discomfort and anxiety/depression [47]. In addition, several aspects with respect to mental functioning, fear of falling and falls are assessed: *mental health and dependency *which is a subscale of the VFQ [41,42], *feelings of anxiety *and *symptoms of depression *as assessed by the Hospital Anxiety and Depression Scale subscales anxiety (HADS-A) and depression (HADS-D) [44,48], *concerns about falling *as assessed by the 16-item Falls Efficacy Scale International (FES-I) [49,50] and a one-item scale [46], as holds as well for *activity avoidance due to fear of falling *[46] and *falls history *indoors and outdoors [46].

With respect to activities frequently present in the O&M-training, being recognised as partially sighted and the use of the identification cane, several questions were formulated (not tabulated). First, related to safety, participants are presented four activities, i.e. crossing a street safely, crossing a major intersection safely, travelling with public transportation and walking in a crowded place. For each of the four activities participants indicate their perceived skills (1 = not at all skilled to 4 = very skilled), their feelings of safety (1 = not at all safe to 4 = very safe) and the frequency in which they perform the activity (1 = never to 4 = almost daily/often). Second, participants indicate how often other people show them consideration in traffic situations and in the city centre or a shop and how often they feel ashamed of their vision loss (1 = very often/often to 4 = never). Lastly, participants indicate to what extent the identification cane is used (1 = never to 4 = almost daily/often) – and, if applicable, in which locations -, how often they expect to use the cane in the upcoming 3 months (1 = never to 4 = almost daily/often), and if applicable, the reason for not using the identification cane.

All primary and secondary outcomes of the effect evaluation were assessed at baseline, and at 5 and 17 week follow-up, except for the outcomes regarding the use of the identification cane. The latter outcomes were solely assessed at 5 and 17 week follow-up. Before randomisation data on descriptive variables and covariates were collected, i.e. with respect to a) the participants: age, gender, marital status, living situation, educational level, employment, year of becoming visually impaired, primary diagnosis, use of an assistive device for mobility purposes, presence of a chronic medical condition (5 major medical conditions were derived from a 19-item checklist [51]), and the problem for which mobility support was requested, and b) the trainers: age, gender, educational level, completion of occupational training, completion of the Dutch national orientation and mobility instruction for mobility trainers, organisation employed, and years of working experience.

#### Process evaluation

To identify programme factors that may influence the effectiveness of the standardised O&M-training and factors that may contribute to future improvement and implementation of the training a process evaluation is carried out. The main outcomes of the process evaluation are derived from previous work [52,53] and Saunders and colleagues [54] and include: population reached, performance according to protocol, exposure and engagement, opinion on the training, perceived benefit or achievement, and experienced barriers and potential solutions. Table 4 provides an overview of the outcomes of the process evaluation during the course of the trial. Data is collected from both the intervention group and the control group.

**Table 4:** Outcome measures of the process evaluation

**Variable**	**Measurement**
**Population reached**	

Target population and proportion of the intended target population	TI-Rp, SQt
General characteristics of the participants and trainers	Qt
Number of participants that refused, dropped out or completed the training	Qt
Reasons for withdrawal	Qt

**Performance according to protocol**	

Format, preparation time and duration of the session	Qt
Per session component: extent to which carried out, duration and active participation by the participant*	Qt
Extent to which the trainer achieved to:	
- related to identification cane: provide information, raise the participant's awareness of its advantages, demonstrate the use and have the participant experience the use of the cane*	Qt
- have the participant phrase his/her important activities related to mobility and how to perform activities safely and independently*	Qt
- have the participant setting goals regarding an action plan*	Qt
- teach orientation skills* and teach mobility skills*	Qt

**Exposure and engagement**	

Total number of sessions	Qt
Use of materials*	TIp, Qt
Overall opinion of the trainer/participant regarding the participant's engagement in:	
- the training	TIp, Qt
- formulating of an action plan and carrying out an action plan*	Qt
Exposure and adherence to homework	TIp
Extent to which the participant complied with contracts*	Qt
Quality of actions plans formulated by the participants*	Qt

**Opinion on the training**	

Overall opinion on the training	TIp, Qt
Opinion regarding:	
- number, duration and course of the sessions	TIp, Qt
- homework and comprehensibility of the complete training	TIp
- number of extra training sessions needed	TIp, Qt
- whether the O&M-training met the participant's need for mobility support	TIp, Qt
Usefulness regarding:	
- discussing mobility problems, receiving information on different kinds of canes and practicing with the identification cane	TIp
- formulating an action plan and carrying out an action plan*	TIp
- contracting with respect to formulating and carrying out action plans*	Tip
Burden experienced by the participant	TIp
Recommendation of the training to others	TIp
Overall opinion on the trainer	TIp, Qt

**Perceived benefit or achievement**	

Benefit regarding:	
- the O&M-training	TIp
- advantages of an identification cane and self-confidence	Qt
- realistic action planning*	Qt
Use of identification cane in daily life	Qt
Achievement regarding:	
- identification cane: knowledge, aware of advantages and having experienced the cane	TIp
- able to think how to perform an activity safely and independently	TIp
- self-confidence and performance of an activity safely and independently	TIp
- formulating a realistic action plan* and carrying out an action plan*	TIp
- skills for orientation and skills for mobility	TIp
- safe and independent participation outdoors	TIp, Qt
- optimal use of personal capabilities	TIp, Qt
- starting new activities and/or taking up former activities that were avoided due to the mobility problems	TIp, Qt

**Experienced barriers and potential solutions**	

Deviations of each session component*	Qt
Main goal of contact - time spent on issues not related to O&M-training	Qt
Strong and weak aspects of the O&M-training	TIp, Qt
Hampering and stimulating factors for use of the standardised O&M-training*	Qt
Matters for improvement - materials and standardised O&M-training*	Qt

* Assessed in the intervention group only.

O&M-training = orientation and mobility training; TI-Rp = telephone interview prior to randomisation with the participant (as part of the screening process); SQt = starting questionnaire, i.e. questionnaire filled in by the trainer before training his/her first participant of the study; Qt = questionnaire filled in by the mobility trainer per participant; TIp = interview by telephone with the participant.

### Data collection

Data for the effect evaluation are collected at baseline, and at 5 and 17 weeks after the start of the O&M-training during 40-minute interviews by telephone. Trained interviewers, who are masked for group allocation, perform the interviews. In addition, a 25-minute interview by telephone is performed at 8 weeks after the start of the O&M-training to obtain data for the process evaluation from participants in the intervention group as well as the control group. Pre-structured questionnaires on process aspects are used to gather data from the mobility trainers per participant. Trainers receive this questionnaire before the first session of the participant's O&M-training. As recommended by Hollis and Campbell [55], non-compliant participants are approached for all follow-up assessments.

### Sample size and power

The power calculation is based on data from Kempen and colleagues [40] with regard to the primary outcome variable: GARS ADL self care. Two times 67 participants will provide 80% power at alpha .05 to detect differences between the intervention and control groups' mean change score of at least 3.5 points (SD is 8.1 equivalent with an effect size of .43 on the GARS ADL self care). However, a dropout during the study of 25% may be expected. Therefore, 2 × 95 participants are needed for randomisation.

### Analysis

Data of the effect evaluation will be analysed according to intention-to-treat principle and on-treatment principle. Univariate techniques and mixed-effects regression analyses will be applied to test for between-group differences with respect to the primary and secondary outcome measures at all follow-up assessments. Models will be adjusted for relevant covariates, including age, gender and baseline differences. Descriptive techniques will be used to describe participants of the trial and to analyse the data of the process evaluation.

## Discussion

O&M-training is a common health care service provided by organisations for rehabilitation care for people with visual impairment. The training aims to maintain independence of travel by teaching visually impaired persons to ambulate and negotiate the environment safely and independently. It may decrease mobility limitations and contribute positively to societal participation and quality of life. In an ageing population it is important to positively influence these domains to prevent physical, mental and social dysfunctioning which can lead to disability and institutionalisation. Yet, evidence on the effects of O&M-training in older adults with visual impairment is scarce, particularly with respect to the O&M-training in the use of an identification cane. This paper presents the design of a randomised controlled trial that will evaluate the effects and feasibility of a standardised O&M-training in the use of the identification cane by older adults with low vision in The Netherlands.

### Progress of the study

The screening procedure for eligible participants started in November 2007 and will continue until October 2009. In April 2009 28 participants (14 control group, 14 intervention group) had completed the training and follow-up assessments and another 11 participants (7 control group and 4 intervention group) had finished the O&M-training and the follow-up assessment at 5 weeks.

Recruitment of the participants by the collaborating organisations Bartiméus and VisioSensisDeBrink Group is slower than expected based on a dossier study by Verstraten and colleagues [56] and information obtained by the first author during interviews with mobility trainers. Reasons for the slow recruitment of participants as stated by the organisations include, among others, lack of eligible clients, lack of interest of clients to participate in the study, other research studies target the same population, work load of the mobility trainers and employees who perform the screening, unwillingness of mobility trainers to wait for the necessary research actions – such as obtaining informed consent and performing an baseline interview – before starting the training, adoption of new computerization system, etc. Although numerous actions have been undertaken to facilitate the organisations in the process of screening and to limit the time for research actions, recruitment of sufficient participants remains the main challenge of this study. The financial budget for the study allows to screen for eligible participants until October 2009. The 2 × 95 participants based on the power calculation will not be enrolled at that time.

Detailed information regarding the development of the standardised O&M-training and the effect and process evaluation will be presented in forthcoming papers. Preliminary findings regarding the evaluation are expected in the course of 2010.

### Future implementation

Throughout the development of the standardised O&M-training, future implementation in the Dutch health care for visually impaired people has been taken into account. An implementation and dissemination plan is designed. One of the main actions consists of embedding the standardised O&M-training in the Dutch national instruction for mobility trainers, i.e. if the standardised O&M-training is more effective than the current O&M-training or, in case of equal effectiveness, is considered more feasible.

## Abbreviations

O&M: training refers to orientation and mobility training; ADL: activities of daily living; IADL: instrumental activities of daily living; ICF: refers to the International Classification of Functioning, Disability and Health.

## Competing interests

The authors declare that they have no competing interests.

## Authors' contributions

GZ, GK, GvR, ES, DB, JvdV and PV contributed to the conception and design of the study. GZ drafted the manuscript with input from the other authors. All authors read and approved the final manuscript.

## Pre-publication history

The pre-publication history for this paper can be accessed here:


